# Parity is associated with albuminuria and chronic kidney disease: a population-based study

**DOI:** 10.18632/aging.102507

**Published:** 2019-12-02

**Authors:** Kan Sun, Diaozhu Lin, Qiling Feng, Feng Li, Yiqin Qi, Chulin Huang, Wanting Feng, Chuan Yang, Li Yan, Meng Ren

**Affiliations:** 1 Department of Endocrinology, Sun Yat-sen Memorial Hospital, Sun Yat-sen University, Guangzhou 510120, People’s Republic of China

**Keywords:** parity, increased urinary albumin excretion, albuminuria, chronic kidney disease, population-based study

## Abstract

Background and aims: Epidemiological studies have shown that increasing parity is associated with risk of hypertension and diabetes in parous women. However, the relationship between the parity degree with chronic kidney disease (CKD) is still unknown.

Results: Parous women with higher parity had increased age, body mass index, waist circumference, systolic blood pressure, fasting plasma glucose, fasting insulin and decreased high-density lipoprotein cholesterol, eGFR and education levels. Compared with women with one-child birth, those with more than two-child births had greater prevalence of increased urinary albumin excretion (odds ratios [ORs] 1.53, 95% confidence intervals [CI], 1.03 - 2.28) and CKD (ORs 1.79, 95% CI, 1.24 - 2.58) after multiple adjustments. In dose-response analysis, a nonlinear relationship of parity degree with albuminuria and CKD was detected.

Conclusion: Parity is associated with higher prevalence of albuminuria and CKD in middle-aged and elderly Chinese women.

Methods: We conducted a community-based study in 6,946 women to investigate the association of parity with albuminuria and CKD. Increased urinary albumin excretion was defined as albumin-to-creatinine ratio (ACR) greater or equal than 30 mg/g. CKD was defined as estimated glomerular filtration rate (eGFR) less than 60 mL/min per 1.73 m² or presence of albuminuria.

## INTRODUCTION

Given the high prevalence rate and the prohibitive spending of maintenance renal replacement therapy, chronic kidney disease (CKD) has become a major public health problem and received increasing attention worldwide [1]. Subjects with CKD are at risk of accelerated cardiovascular disease and related death, even in the early stage. Screening for individuals at high risk of renal impairment is an effective strategy for CKD management.

Several epidemiological studies have shown that increasing parity may affect the health of women later in life. Vaidya et al. [2] reported a strong association of parity history with lower carotid artery distensibility. Their findings suggested that arterial remodeling lasts beyond giving birth, which may explain the association of parity with cardiovascular events in later life. It is reported that parity degree was associated with increased risk of metabolic syndrome in women aged 50 years or older [3]. Recent studies also indicated that parity degree is independently associated with increased risk of both hypertension and diabetes, which will result in a greater burden of CKD among parous women [4, 5]. Compared with non-pregnant values, GFR was reduced in early pregnancy but increased significantly during the final trimester of pregnancy due to the volume expansion in the stage [6]. Consequently, in women with previous pre­existing kidney damage, glomerular hyper-filtration in the last trimester of pregnancy would go beyond the scope of renal compensation and trigger rapid progression of nephropathy [1]. In addition, multiparous women had marked fluctuation in estrogen and other anti-insulin hormones, and could promote the development and pathogenesis of kidney damage [7–10].

In light of the above perspectives, we assumed that parity degree may have potential effect on the presence and progression of renal impairment. However, little is known concerning the association between parity degree and CKD. Therefore, in the present study, we analyzed data from a Chinese population to explore the possible association of parity with albuminuria and CKD.

## RESULTS

### Clinical characteristics of the study population

The mean age was 55.2 ± 7.7 years among the 6,946 enrolled women. In total, 64.6% (4,489) of women were one-child birth and 12.5% (869) of women were nulliparous. There were 451 (6.5%) subjects categorized as increased urinary albumin excretion and 511 (7.4%) subjects categorized as CKD, respectively. Table 1 shows the clinical and biochemical characteristics of the participants according to parity degree. Parous women with higher parity number had increased age, BMI, WC, SBP, FPG, fasting insulin, and decreased HDL-C, eGFR and education levels. Compared with women giving one live birth in their life, nulliparous women were older and had higher TC, LDL-C, γ-GGT, proportions of current smokers, proportions of current drinkers and decreased eGFR levels.

**Table 1:** Characteristics of study population by parity degree.

	**Number of Parity**			
	**0**	**1**	**2**	**≥ 3**
n (%)	869 (12.5)	4489 (64.6)	1069 (15.4)	519 (7.5)
Urinary ACR (mg/g)	8.25 (5.92 – 12.25)	8.33 (5.97 – 12.19)	9.16 (6.51 – 13.73)^#, &^	10.46 (6.66 – 16.32)^#, &^
Age (years)	54.4 ± 7.2^#^	53.4 ± 5.7^&^	58.2 ± 8.8^#, &^	65.6 ± 10.9^#, &^
BMI (kg/m^2^)	23.3 ± 3.3	23.4 ± 3.4	24.2 ± 3.3^#, &^	24.6 ± 3.8^#, &^
WC (cm)	79.5 ± 10.0	79.5 ± 8.8	82.6 ± 9.3^#, &^	84.9 ± 9.4^#, &^
SBP (mmHg)	124.2 ± 15.6	123.4 ± 15.8	128.2 ± 16.9^#, &^	133.0 ± 17.4^#, &^
DBP (mmHg)	74.4 ± 9.3	74.1 ± 9.6	75.0 ± 9.7^#^	74.7 ± 9.7
Current smoking [n (%)]	22 (2.8)^#^	42 (1.0)^&^	12 (1.1)^&^	11 (2.1)^#^
Current drinking [n (%)]	15 (2.1)^#^	48 (1.1)^&^	17 (1.6)	6 (1.2)
TG (mmol/L)	1.28 (0.94 – 1.81)^#^	1.20 (0.88 – 1.71)^&^	1.31 (0.98 – 1.86) ^#^	1.44 (1.00 – 2.01) ^#, &^
TC (mmol/L)	5.44 ± 1.25^#^	5.24 ± 1.28^&^	5.25 ± 1.27	5.23 ± 1.26^&^
HDL-C (mmol/L)	1.41 ± 0.36	1.38 ± 0.37	1.35 ± 0.35^&^	1.28 ± 0.34^#, &^
LDL-C (mmol/L)	3.26 ± 0.96^#^	3.16 ± 0.98^&^	3.17 ± 0.96	3.15 ± 0.95
FPG (mmol/L)	5.37 (4.94 – 5.88)	5.35 (4.96 – 5.81)	5.51 (5.07 – 6.03)^#, &^	5.63 (5.14 – 6.20)^#, &^
Fasting insulin (μIU/ml)	7.20 (5.20 – 9.90)	7.10 (5.30 – 9.80)	7.90 (5.90 – 11.00)^#, &^	8.20 (5.90 – 11.30)^#, &^
γ-GGT (U/L)	19.0 (14.0 – 27.0)^#^	17.0 (13.0 – 25.0)^&^	19.0 (14.0 – 27.0)	19.0 (14.0 – 26.0)
eGFR (ml/min per 1.73 m^2^)	102.9 ± 23.0^#^	105.9 ± 24.2^&^	102.1 ± 21.4^#^	96.5 ± 23.0^#, &^
Physical activity (MET-h/week)	10.5 (0.0 – 36.0)	25.0 (12.0 – 49.0)	28.0 (12.0 – 49.0)	21.0 (10.5 – 42.0)
Spontaneous abortion [n (%)]	30 (3.5)^#^	279 (6.2)^&^	76 (7.1)^&^	63 (12.1)^#, &^
Menopause [n (%)]	431 (75.0)	3247 (73.4)	847 (80.4)^#, &^	456 (88.2)^#, &^
High school or higher education [n (%)]	490 (70.8)	3057 (68.7)	399 (37.9) ^#, &^	62 (12.1) ^#, &^

1. Data were means ± SD or medians (interquartile ranges) for skewed variables or numbers (proportions) for categorical variables; P values were for the ANOVA or χ^2^ analyses across the groups.

2. ^#^ P < 0.05 compared with participants with one live birth (parity number = 1 group); ^&^ P < 0.05 compared with participants with no live birth (parity number = 0 group).

3. ACR, albumin to creatinine ratio; BMI, body mass index; WC, waist circumference; SBP, systolic blood pressure; DBP, diastolic blood pressure; TG, triglycerides; TC, total cholesterol; HDL-C, high-density lipoprotein cholesterol; LDL-C, low-density lipoprotein cholesterol; FPG, fasting plasma glucose; γ-GGT, γ-glutamyltransferase; eGFR, estimated glomerular filtration rate.

### Associations of parity degree with increased urinary albumin excretion and CKD

**Figure 1:**
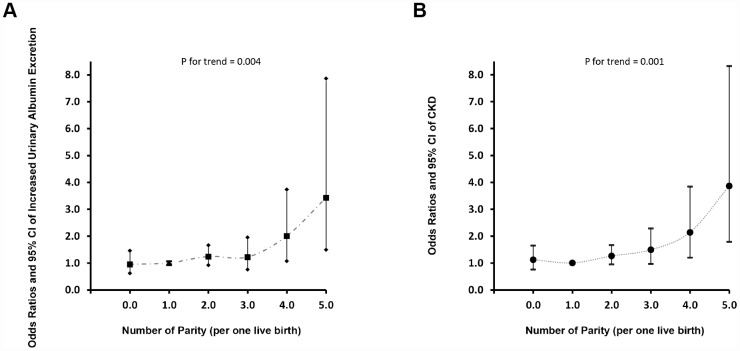
**Dose-response analyses of parity number with increased urinary albumin excretion and CKD.** (**A**) Increased Urinary Albumin Excretion; (**B**) CKD.

Among parity number in 0, 1, 2 and ≥ 3 groups, the prevalence of increased urinary albumin excretion were 5.6%, 5.6%, 8.2% and 11.8% while the prevalence of CKD were 6.9%, 6.0%, 9.5% and 15.2%, respectively (Figure 1A and 1B). Compared with women with one-child birth (parity number = 1), subjects with more than 2 live births (parity number ≥ 3) were independently associated with a greater prevalence of increased urinary albumin excretion (ORs 1.53, 95% CI, 1.03 - 2.28) and CKD (ORs 1.79, 95% CI, 1.24 - 2.58) in multivariate logistic regression analyses (Table 2). When compared nulliparous women (parity number = 0) with women with one-child birth (parity number = 1), no statistically significant difference of such associations was found.

**Table 2:** The risk of prevalent increased urinary albumin excretion and CKD according to parity degree.

		**Number of Parity**			
		**0**	**1**	**2**	**≥ 3**
Increased urinary albumin excretion	Model 1	1.00 (0.73 – 1.37)	1	1.50 (1.17 – 1.93)	2.23 (1.66 – 3.00)
	Model 2	0.99 (0.72 – 1.35)	1	1.42 (1.09 – 1.84)	1.91 (1.36 – 2.69)
	Model 3	0.96 (0.63 – 1.47)	1	1.23 (0.92 – 1.65)	1.55 (1.06 – 2.28)
	Model 4	0.95 (0.62 – 1.45)	1	1.22 (0.91 – 1.64)	1.53 (1.03 – 2.28)
CKD	Model 1	1.15 (0.86 – 1.54)	1	1.62 (1.28 – 2.06)	2.80 (2.14 – 3.66)
	Model 2	1.12 (0.84 – 1.50)	1	1.42 (1.10 – 1.82)	2.10 (1.53 – 2.88)
	Model 3	1.13 (0.77 – 1.66)	1	1.26 (0.95 – 1.66)	1.86 (1.30 – 2.64)
	Model 4	1.11 (0.75 – 1.64)	1	1.25 (0.94 – 1.66)	1.79 (1.24 – 2.58)

Data are odds ratios (95% confidence interval). Participants without increased urinary albumin excretion or CKD are defined as 0 and with increased urinary albumin excretion or CKD as 1.

Model 1 is unadjusted.

Model 2 is adjusted for age.

Model 3 is further adjusted for BMI, current smoking status, current drinking status, education levels and physical activity.

Model 4 is further adjusted for SBP, TG, LDL-C, FPG and fasting insulin.

### Subgroups and dose-response analysis of parity degree with increased urinary albumin excretion and CKD

**Figure 2:**
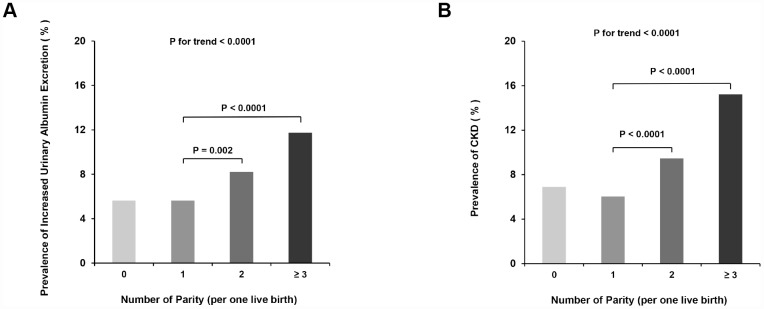
**Prevalence of increased urinary albumin excretion and CKD according to elevated parity degree.** (**A**) Increased Urinary Albumin Excretion; (**B**) CKD.

The associations of parity degree with increased urinary albumin excretion and CKD were inconsistent in subgroups analyses (Table 3). In overweight and no spontaneous abortion history subgroup, women with more than 2 live births were independently associated with a greater prevalence of increased urinary albumin excretion and CKD when compared with those with one-child birth. In dose-response analysis, nonlinear associations of parity degree with both increased urinary albumin excretion and CKD were found and higher parity number seems to remarkably increased prevalent albuminuria and CKD (Figure 2A and 2B).

**Table 3:** Association of prevalent increased urinary albumin excretion and CKD with parity degree in different subgroups.

		**Number of Parity**			
		**0**	**1**	**2**	**≥ 3**
Increased urinary albumin excretion	BMI				
	Normal	0.90 (0.49 – 1.64)	1	1.08 (0.67 – 1.72)	1.72 (0.92 – 3.20)
	Overweight	0.90 (0.44 – 1.87)	1	1.05 (0.66 – 1.67)	2.04 (1.15 – 3.59)
	Obesity	1.38 (0.41 – 4.70)	1	2.29 (1.10 – 4.75)	0.32 (0.09 – 1.17)
	Spontaneous abortion				
	Yes	2.28 (0.43 – 12.02)	1	0.92 (0.23 – 3.73)	2.49 (0.59 – 10.51)
	No	0.90 (0.58 – 1.40)	1	1.27 (0.94 – 1.71)	1.53 (1.01 – 2.31)
	Menopause				
	Yes	1.04 (0.63 – 1.71)	1	1.10 (0.78 – 1.54)	1.23 (0.78 – 1.95)
	No	0.81 (0.31 – 2.13)	1	1.39 (0.73 – 2.66)	2.01 (0.77 – 5. 25)
	eGFR (ml/min per 1.73 m^2^)				
	eGFR ≥ 90	0.74 (0.42 – 1.31)	1	1.27 (0.90 – 1.79)	1.29 (0.77 – 2.17)
	90 > eGFR ≥ 60	1.59 (0.78 – 3.24)	1	1.19 (0.64 – 2.20)	2.45 (1.18 – 5.09)
	eGFR < 60	0.19 (0.01 – 3.55)	1	0.25 (0.03 – 2.07)	0.01 (0.00 – 0.40)
CKD	BMI				
	Normal	1.03 (0.60 – 1.78)	1	1.03 (0.66 – 1.62)	2.27 (1.30 – 3.98)
	Overweight	1.22 (0.64 – 2.34)	1	1.17 (0.76 – 1.83)	2.09 (1.21 – 3.61)
	Obesity	1.23 (0.36 – 4.17)	1	2.21 (1.09 – 4.50)	0.42 (0.13 – 1.37)
	Spontaneous abortion				
	Yes	2.18 (0.42 – 11.44)	1	0.98 (0.27 – 3.59)	2.52 (0.64 – 9.98)
	No	1.08 (0.72 – 1.61)	1	1.28 (0.96 – 1.71)	1.79 (1.22 – 2.63)
	Menopause				
	Yes	1.02 (0.63 – 1.65)	1	1.16 (0.84 – 1.60)	1.59 (1.04 – 2.43)
	No	0.81 (0.31 – 2.13)	1	1.39 (0.73 – 2.66)	2.01 (0.77 – 5.25)

Data are odds ratios (95% confidence interval). Participants without increased urinary albumin excretion or CKD are defined as 0 and with increased urinary albumin excretion or CKD as 1. Odds ratios were adjusted for age, BMI, current smoking status, current drinking status, education levels, physical activity, SBP, TG, LDL-C, FPG and fasting insulin.

## DISCUSSION

In summary, we found that parity degree is associated with higher prevalence of albuminuria and CKD among Chinese women aged 40 years or older. To the best of our knowledge, this is the first population-based study to reveal associations of parity degree with prevalent albuminuria and CKD.

Pregnancy has a long-term influence on women’s health that result from the remarkable metabolic alterations in endocrine and immune system. In the field of kidney disease, only one study has reported on the relationship between parity and risk of death from chronic renal failure (CRF) at the present [11]. In that study, parity may have protective effect on the risk of death from CRF. However, in this particular study, there were only 225 CRF deaths during 34,980,246 person-years of follow-up. As data on the accuracy of CRF diagnosis is unavailable, the potential for misclassification of CRF is one serious limitation of the study. In fact, high parity degree is often cited as adverse risk factors for metabolic related diseases [3–5]. The finding of present study indicated that parity degree, as a risk factor for hypertension and diabetes, is associated with albuminuria and CKD in a dose-response manner, which should be considered in early prevention of renal disease.

Subgroup analysis of the association between parity degree and CKD highlights the importance of paying attention to BMI categories in multiparous women. In BMI < 28 subgroups (normal and overweight women), we found that higher parity degree was significantly associated with increasing prevalent CKD. As BMI ≥ 28 (obese) resulted in greater-grade manifestation of adipose tissue accumulation, it is likely that there are more unmeasured metabolic risk factors in this subgroup, some of which might correlate with parity degree and dilute the main findings of this study. Another possible reason for the inconsistent effect in BMI categories is that women who had more children and currently obese were general healthier than those who had less children, and also they had less risk of prevalent CKD. However, the underlying mechanism remains unexplained and requires further exploration. Seneviratne et al. [12] have recently reported that nulliparous and multiparous women may confer subtle metabolic disadvantage in overweight and obese mothers and their offspring. In our study, nulliparous women and women with two live birth may have similar magnitude of association with CKD, although the associations were not statistical significant. Therefore, caution should be taken in interpreting the results among these subgroups, especially in nulliparous overweight and obese women. Moreover, controversy remains regarding the underlying disease in nulliparous women. Possible conditions such as autoimmune and endocrine diseases were associated with albuminuria and CKD, which could preclude women from pregnancy or childbearing. Longitudinal studies performed through excluding such preexisting diseases will be necessary to verify the present findings in nulliparous women.

Mechanism responsible for elevated CKD risk caused by parity remains uncertain and some biologic hypotheses have been proposed. The balance between matrix metalloproteinases (MMP) and tissue inhibitors of metalloproteinases (TIMP) played a crucial role in degradation and remodeling of extracellular matrix and angiogenesis [13, 14]. During pregnancy, abnormalities of the matrix metalloproteinase system could result in hypoxia, oxidative stress, inflammatory response and proteinuria, which may be involved in the pathophysiology of systemic renal arteries remodeling [15–17]. Insulin resistance correlated linearly with decline in renal function and occurs in patients with CKD at different stages of kidney impairment [18, 19]. The levels of various anti-insulin hormones increased in fertile women and involved in promoting insulin resistance and the pathophysiology of arterial hypertension and angiogenesis [10, 20]. Accordingly, long lasting exposure to high levels of anti-insulin hormones during the multiple parity may therefore as a risk factor linking decreased renal function.

The study had several limitations that require further consideration. First, all clinical and biochemical measurements were collected at the same time of outcome measurement. Hence, owing to the nature of observational design of the current study, we should be cautious regarding the interpretation of whether parity is a causal factor of increased urinary albumin excretion and CKD. Second, the creatinine alteration present for more than 3 months is a part of the CKD definition. However, we evaluated serum creatinine and urinary albumin excretion on the basis of a single measurement, which may result in overestimation of the prevalence of CKD. Third, the original population of the present study is a female dominant population. Partially because participants in baseline the survey were all over 40 years and females were accounted for most proportion in this age range. Fourth, disorders during the pregnancies or abnormal obstetrical outcomes, i.e., preeclampsia, preterm labor, gestational diabetes mellitus and acute kidney injury are related to progressive renal function impairment. Preeclampsia is one of the challenge during pregnancy, which could cause transitory kidney damage and increased risk of developing CKD later in life [21, 22]. Moreover, acute kidney injury (AKI) in pregnancy is often caused by preeclampsia or eclampsia [23]. Although most patients with AKI are reported to have renal function recovery, more than 10% of these patients will still progress to CKD during follow-up [24, 25]. Missing of these data may influence result interpreting in the study. Fifth, incomplete data compilation may influence the interpretation of the result of this study. Therefore, more detailed information of previous nephropathy history, salt consumption, personal income levels and nutrition and lifestyle in pregnancy should be considered to collect to strength the findings of the present study.

In conclusion, our study highlights the importance of paying more attention to early kidney damage in women with higher frequency of pregnancy. Further prospective studies are necessary to verify our findings in other countries and ethnic groups.

## MATERIALS AND METHODS

### Study population and design

The study population was taken from the Risk Evaluation of Cancers in Chinese Diabetic Individuals: A Longitudinal Study (the REACTION Study), and details of this cohort have been published previously [26–28]. Baseline survey of the REACTION study recruited nearly 260,000 subjects from 25 communities across mainland China between the year 2011 and 2012. The communities were selected from different regions in both urban and rural sites, with distinct degrees of economic and urbanization development status. All individuals with self-care ability aged 40 years and older were allowed to participate in the baseline survey and included in the study.

**Figure 3:**
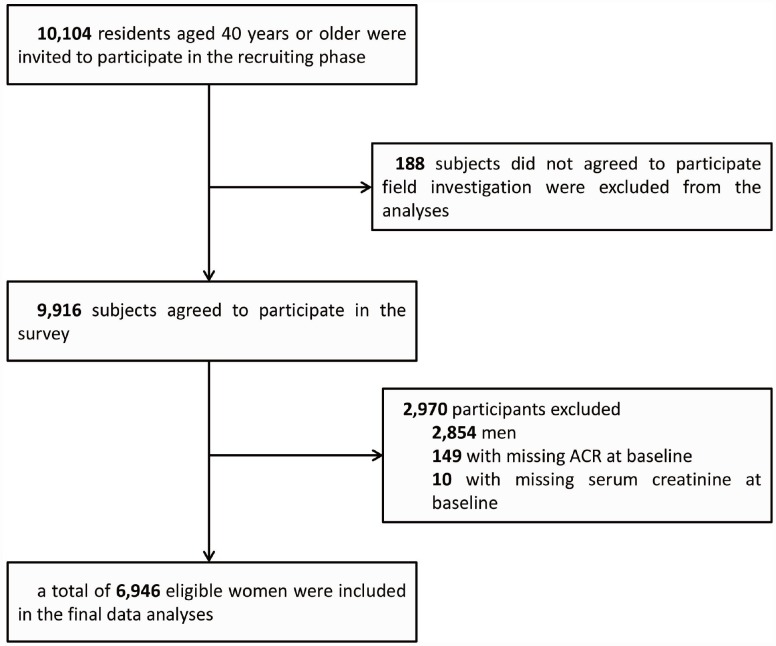
**Flowchart of the selection of the study participant.**

We performed the present retrospective study in one of the centers from June to November, 2011 for the community located in Guangzhou, China. During the recruiting phase, a total of 10,104 residents aged 40 years or older were invited to participate by examination notices or home visits. In total, 9,916 subjects agreed to participate in the survey, and the participation rate was 98.1%. In data analyses, men (n = 2,854) were first excluded from the study. Subjects who failed to provide information (urinary albumin-to-creatinine ratio [ACR]: n = 149; serum creatinine: n = 10) were then excluded from the analyses. Accordingly, a total of 6,946 eligible women were included in the final data analyses. The details of the selection of study participants are presented in a flow diagram (Figure 3).

The study protocol was approved by the Institutional Review Board of the Sun Yat-sen Memorial Hospital, Sun Yat-sen University and was in accordance with the principle of the Helsinki Declaration II. Written informed consent was obtained from each participant before data collection.

### Questionnaire investigation

Through a detailed questionnaire, we collected information on lifestyle factors and sociodemographic characteristics of each participant. Information on reproductive history was self-reported. Women were asked to recall the number of pregnancies and parity. Smoking or drinking habit was classified as ‘never’, ‘current’ (smoking or drinking regularly in the past 6 months) or ‘ever’ (cessation of smoking or drinking more than 6 months) [29]. A short form of the International Physical Activity Questionnaire (IPAQ) was used to estimate physical activity at leisure time by adding questions on frequency and duration of moderate or vigorous activities and walking [30]. Separate metabolic equivalent hours per week (MET-h/week) were calculated for evaluation of total physical activity. Education levels were categorized as less than middle school, middle school graduate and high school graduate or higher.

### Clinical and biochemical measurements

All participants completed anthropometrical measurements with the assistance of trained staff by using standard protocols. Three consecutive blood pressure measurements were obtained within a 5-minute interval with automated electronic device (OMRON, Omron Company, China). The average of three measurements of blood pressure was used for analysis. Body height and weight were recorded to the nearest 0.1 cm and 0.1 kg, respectively. Participants were required to remove their shoes and wear light indoor clothing during the measurement. BMI was calculated as weight in kilograms divided by height in meters squared (kg/m^2^). Reference to the local population prevalence of obesity and for the BMI threshold was used to define obesity in the study [31–34]. Accordingly, obesity was defined as BMI ≥ 28 and overweight was defined as BMI ≥ 24 and < 28. WC was measured at the umbilical level with participant in standing position, at the end of gentle expiration. Venous blood samples were collected for laboratory tests after an overnight fasting of at least 10 hours. Measurement of fasting plasma glucose (FPG), fasting serum insulin, triglycerides (TG), total cholesterol (TC), high-density lipoprotein cholesterol (HDL-C), low-density lipoprotein cholesterol (LDL-C), creatinine, and γ-glutamyltransferase (γ-GGT) was done using an autoanalyser (Beckman CX-7 Biochemical Autoanalyser, Brea, CA, USA).

### Definition of increased urinary albumin excretion and CKD

The abbreviated Modification of Diet in Renal Disease (MDRD) formula recalibrated for Chinese population was used to calculate estimated glomerular filtration rate (GFR) expressed in ml/min per 1.73 m^2^ using a formula of eGFR = 175 × [serum creatinine × 0.011]^-1.234^ × [age]^-0.179^ × [0.79 if female], where serum creatinine was expressed as μmol/L [35]. Definitions of albuminuria were according to the latest guidelines of American Diabetes Association’s Standards of Medical Care [36]. The first morning spot urine samples were collected for assessing the ACR. Urine albumin and creatinine were measured by chemiluminescence immunoassay (Siemens Immulite 2000, United States) and the Jaffe’ s kinetic method (Biobase-Crystal, Jinan, China) on the automatic analyzer, respectively. ACR was calculated by dividing the urinary albumin concentrations by the urinary creatinine concentrations and expressed in mg/g. The outcome measures of the study were increased urinary albumin excretion and CKD. Increased urinary albumin excretion was defined according to the ACR ranges greater or equal than 30 mg/g. Chronic kidney disease (CKD) was defined as eGFR less than 60 mL/min per 1.73 m² or presence of albuminuria (ACR greater or equal than 30 mg/g) [37].

### Statistical analysis

Continuous variables were presented as means ± standard deviation (S.D.) except for skewed variables, which were presented as medians (interquartile ranges). Categorical variables were expressed as numbers (proportions). FPG, fasting insulin, TG, γ-GGT and MET-h/week were logarithmically transformed before analysis due to a non-normal distribution. Differences among groups were tested by one-way ANOVA and *post hoc* comparisons were performed by using Bonferroni correction. Comparisons between categorical variables were performed with the χ^2^ test.

We analyzed the impact of parity on the prevalence of increased urinary albumin excretion and CKD. The unadjusted and multivariate-adjusted logistic regression analysis was used to assess the risk of prevalent increased urinary albumin excretion and CKD in relation to degree of parity. The covariates included in the multivariate-adjusted logistic regression analysis were selected based on previous publications and potential risk factors that associated with the progression of renal disease [37–39]. Model 1 is unadjusted. Model 2 is adjusted for age. Model 3 is further adjusted for BMI, current smoking status, current drinking status, education levels and physical activity. Model 4 is further adjusted for SBP, TG, LDL-C, FPG and fasting insulin. Odds ratios (ORs) and the corresponding 95% confidence intervals (95% CI) were calculated. In all logistic regression analysis, test for linear trend across groups was treating number of parity as a continuous variable. Relationship of parity with albuminuria and CKD in model 4 of logistic regression analysis was explored in subgroups stratified degree of obesity (normal/ overweight/ obese), spontaneous abortion history (yes/ no) and menopause (yes/ no). For relationship between parity and albuminuria, subgroup analysis stratified by eGFR level (≥ 90; 60-89; < 60 ml/min per 1.73 m^2^) was also conducted. The dose-response relationship between parity and prevalent increased urinary albumin excretion and CKD were further performed by using multivariate adjusted logistic regression analyses in model 4. In dose-response analysis, number of parities were classified as follows: 0, 1, 2, 3, 4 and ≥ 5 (per one live birth).

All statistical analysis was performed using SAS version 9.3 (SAS Institute Inc, Cary, NC, USA). Statistical tests were two-sided, and a P value < 0.05 was considered statistically significant.

### Ethical standards

Protocol of the present study involved human participant was approved by the Institutional Review Board of the Sun Yat-sen Memorial Hospital, Sun Yat-sen University and was in accordance with the principle of the Helsinki Declaration II. We obtained written informed consent with permission to use the data from each participant before data collection.
